# Continuous Glucose Monitoring for Metabolic Phenotyping and Precision Nutrition: Current Evidence, Clinical Applications and Future Perspectives

**DOI:** 10.3390/diagnostics16203415

**Published:** 2026-10-20

**Authors:** Liora Colobatiu, Romolica Mihaiu, Alexandra Pusta, Simona Mirel

**Affiliations:** 1 Department of Medical Devices, Faculty of Pharmacy, Iuliu Hatieganu University of Medicine and Pharmacy, 400349 Cluj-Napoca, Romania; mihaela.colobatiu@umfcluj.ro (L.C.); smirel@umfcluj.ro (S.M.); 2 Department of Management, Faculty of Economics and Business Administration, Babes-Bolyai University, 400591 Cluj-Napoca, Romania; romolica.mihaiu@econ.ubbcluj.ro

**Keywords:** continuous glucose monitoring, precision nutrition, metabolic phenotyping, postprandial glycemic response, glycemic variability, personalized nutrition, machine learning, gut microbiome, wearable biosensors

## Abstract

Continuous glucose monitoring (CGM) has evolved beyond conventional diabetes management into an enabling technology for metabolic phenotyping and precision nutrition by providing continuous, real-world assessment of individual glycemic responses to dietary and lifestyle exposures. This narrative review examines the principles and clinical utility of CGM and its emerging applications in metabolic monitoring and personalized nutrition, with particular emphasis on postprandial glycemic variability, computational prediction, dietary feedback, and behavioral interventions. A literature search was conducted in Web of Science, PubMed, Scopus, and Google Scholar using predefined combinations of terms related to CGM, precision and personalized nutrition, diet, postprandial glycemic responses, lifestyle, glycemic variability, artificial intelligence, machine learning, and computational nutrition, including English-language literature available up to June 2026. Current evidence demonstrates substantial interindividual variability in postprandial glycemic responses and supports the potential integration of CGM with dietary, clinical, anthropometric, behavioral, and microbiome-derived data to characterize individual metabolic responses and inform individualized prediction and nutritional guidance. However, the reproducibility and generalizability of CGM-derived metabolic phenotypes and predictive models remain incompletely established. CGM-supported nutritional and behavioral interventions have demonstrated improvements in glycemic and selected cardiometabolic outcomes, particularly in individuals with type 2 diabetes, whereas evidence of clinical benefit in populations without diabetes remains substantially more limited. Sustained engagement and psychological, technical, and accessibility-related limitations also remain important considerations. Emerging multi-analyte sensing technologies may further broaden continuous metabolic assessment and support more comprehensive, data-driven precision-nutrition strategies.

## 1. Introduction

Suboptimal dietary habits represent one of the leading threats to global health, contributing substantially to the worldwide burden of chronic disease. Large-scale international analyses have demonstrated that poor diet accounts for approximately one in five deaths globally, exceeding the mortality attributable to several other major risk factors, including tobacco use [1]. Consequently, improving dietary quality has become a central objective in strategies aimed at preventing and managing non-communicable diseases [1,2]. Among these conditions, type 2 diabetes mellitus and prediabetes continue to increase in prevalence, exposing millions of individuals to an elevated risk of both macrovascular and microvascular complications. Persistent hyperglycemia contributes to endothelial dysfunction and irreversible tissue damage, leading to complications such as diabetic retinopathy, nephropathy, neuropathy, and neurovascular degeneration. Beyond their clinical consequences, these disorders impose a substantial economic burden on healthcare systems worldwide [3,4,5,6,7,8]. Therefore, effective monitoring of dietary intake and eating behavior represents an essential component of metabolic disease prevention and management. However, conventional dietary assessment methods, including food diaries and 24-hour dietary recalls, rely predominantly on self-reporting, making them vulnerable to recall bias, inaccurate estimation of portion sizes, low long-term adherence, and systematic misreporting of energy and macronutrient intake [1,9,10,11]. Traditional nutritional recommendations have largely been based on population-level approaches that assume relatively uniform metabolic responses across individuals. Increasing evidence, however, has challenged this “one-size-fits-all” paradigm by demonstrating considerable interindividual variability in glycemic responses to identical foods. Foods that produce minimal glycemic variations in one individual may elicit pronounced glucose elevations in another, reflecting the complex interaction of multiple biological, environmental, and behavioral determinants [12,13,14,15]. Individual postprandial metabolic responses are influenced by a broad network of factors, including genetic background, epigenetic regulation, gut microbiota composition, phenotypic characteristics, physical activity, sleep, circadian rhythms, psychological stress, and numerous meal-related variables such as food composition, preparation methods, meal timing, and food sequencing [16,17,18]. These observations have accelerated the transition towards precision nutrition, an approach that integrates individual biological, environmental, and behavioral characteristics to develop targeted nutritional strategies for disease prevention, treatment, and health optimization. According to the International Society for Nutrigenetics and Nutrigenomics, personalized nutrition encompasses progressively increasing levels of individualization, ranging from population-based recommendations to phenotype-driven interventions and, ultimately, highly personalized dietary strategies informed by rare genetic variants with large biological effects [16,17,19]. CGM has emerged as an enabling technology supporting this transition towards precision nutrition [20,21]. CGM systems employ minimally invasive sensors inserted into the subcutaneous tissue of the upper arm or abdomen, to continuously measure glucose concentrations within the interstitial fluid [22,23]. Initially developed primarily for insulin management in individuals with type 1 diabetes and insulin-treated type 2 diabetes, CGM has recently expanded beyond traditional therapeutic applications [24]. Reduced device costs, regulatory approval of over-the-counter systems, and integration with smartphone-based digital health platforms have facilitated adoption among individuals with prediabetes, obesity, athletes, and health-conscious consumers without diabetes, including those interested in metabolic health and personalized lifestyle assessment. However, this expanding use should be distinguished from established clinical utility. Although CGM can characterize postprandial glucose dynamics and glycemic variability in individuals without diabetes, the clinical significance of these fluctuations is less clearly defined, and current evidence supporting CGM-guided lifestyle or nutritional interventions in these populations remains limited compared with that available in diabetes. Accordingly, the ability of CGM to identify individual glycemic responses should not, by itself, be interpreted as evidence that these responses represent clinically actionable abnormalities or that modifying them improves long-term health outcomes [25,26,27,28].

A major advantage of CGM lies in its ability to provide continuous physiological biofeedback by displaying glucose dynamics throughout the day. Real-time access to these data enables individuals to directly observe glucose responses in relation to dietary choices and lifestyle behaviors, thereby promoting active engagement in metabolic self-management [24,29]. Rather than relying solely on retrospective clinical measurements, CGM allows assessment of postprandial glycemic response (PPGR), which represents the immediate glucose excursion following food consumption [17]. Although dietary carbohydrates constitute the principal determinant of postprandial glucose elevations, the addition of dietary fiber, protein, and healthy fats substantially modifies the magnitude and duration of the glycemic response [1,30]. In contrast to conventional biomarkers such as fasting glucose and glycated hemoglobin (HbA1c), which provide static estimates of glycemic status, CGM captures dynamic glucose patterns and postprandial responses that can contribute to individual metabolic phenotyping and support individualized nutritional assessment and precision-nutrition strategies [24,31]. Metabolic phenotyping by CGM provides an extensive analysis of glycemic profiles that can help group individuals by “glucotypes” and indicates that even normoglycemic individuals can experience high glucose variability [32].

The increasing availability of continuous glucose data has also contributed to the emergence of computational nutrition, an interdisciplinary field integrating data science, statistics, epidemiology, computer science, systems biology, and clinical medicine to develop personalized nutritional recommendations [16]. Machine learning approaches have demonstrated the ability to predict PPGR by combining clinical, anthropometric, behavioral, and microbiome data, with subsequent validation studies confirming the reproducibility of these predictive models in independent populations [12,16,17]. More recently, computational approaches have progressed towards solving the inverse metabolic problem, whereby the macronutrient composition of consumed meals is inferred directly from postprandial glucose curves [1,2]. Simultaneously, technological development is expanding towards integrated multi-analyte wearable systems capable of monitoring additional biomarkers, including ketones, micronutrients, metabolites, and salivary analytes, thereby extending the scope of personalized metabolic assessment [1]. Growing clinical evidence further supports the integration of CGM into lifestyle and nutritional interventions, where continuous access to glucose data provides physiological feedback that can encourage modifications in food selection, portion size and physical activity [7,29,33]. CGM-supported nutritional and behavioral interventions have been associated with improvements in glycemic control and selected cardiometabolic outcomes [24,29,31]. Nevertheless, broader implementation remains subject to psychological, economic, and technical limitations, including CGM-related distress and data fatigue, device cost and limited reimbursement, as well as limitations related to sensor performance [16,19,34]. Against this background, the present narrative review examines CGM as an enabling technology for metabolic phenotyping and precision nutrition. It provides an overview of the principles, analytical performance, and clinical utility of CGM and examines its role in characterizing individualized postprandial glycemic responses. Particular attention is given to the integration of CGM-derived data with dietary, clinical, anthropometric, behavioral, and microbiome-derived information through computational approaches, and to the potential translation of these data into individualized nutritional guidance. The review further evaluates clinical evidence from CGM-supported nutritional and behavioral interventions, addresses the technical, psychological, and accessibility-related limitations associated with broader implementation, and discusses emerging sensing technologies that may extend continuous metabolic monitoring beyond glucose.

## 2. Materials and Methods

This study was conducted as a structured narrative review examining continuous glucose monitoring (CGM) as an enabling technology for metabolic phenotyping and precision nutrition. The literature search was conducted using Web of Science, PubMed, Scopus, and the academic search engine Google Scholar. The following keyword combinations were used: continuous glucose monitoring AND precision nutrition, continuous glucose monitoring AND personalized nutrition, continuous glucose monitoring AND diet, continuous glucose monitoring AND postprandial glycemic response, continuous glucose monitoring AND lifestyle, continuous glucose monitoring AND glycemic variability, continuous glucose monitoring AND artificial intelligence, continuous glucose monitoring AND machine learning, and continuous glucose monitoring AND computational nutrition. The search included English-language publications available up to June 2026, with no restriction on publication date or study design.

Retrieved publications were screened for relevance to the scope of the review based on titles and abstracts, followed by full-text assessment where appropriate. Publications were considered relevant when they addressed CGM principles, analytical performance, or clinical utility; characterization or prediction of postprandial glycemic responses; CGM-supported nutritional or behavioral interventions; integration of CGM data with dietary, clinical, anthropometric, behavioral, or microbiome-derived information; psychological or implementation-related considerations; or emerging technologies relevant to continuous metabolic monitoring. Studies involving different populations were considered where relevant to the scope of the review. However, evidence from individuals with diabetes and populations without diabetes was interpreted separately when differences in the extent and clinical relevance of the available evidence were important.

Given the heterogeneity of the literature and the narrative design of the review, evidence was synthesized qualitatively rather than through a formal meta-analysis or systematic risk-of-bias assessment. When interpreting clinical intervention studies, particular attention was given to study design, population and sample size, follow-up duration, comparator, intervention composition, and methodological limitations. For the studies summarized in Table 1, these characteristics were considered together with the main reported outcomes and available information on funding and conflicts of interest. This approach allowed evidence from systematic reviews and meta-analyses, randomized studies, and uncontrolled pilot studies to be distinguished according to study design and limitations, particularly when CGM formed part of a broader nutritional or behavioral intervention.

During revision, targeted supplementary searches and checks of primary and authoritative sources were performed for specific areas requiring further clarification, including CGM use in populations without diabetes, microbiome-related mechanisms, psychological effects, emerging sensing technologies, regulatory and data-protection considerations, and CGM performance standardization. References supporting quantitative findings and key statements were also re-checked against the corresponding source publications. As the review was not prospectively designed or registered as a systematic review, exact record-level counts for identification, duplicate removal, screening, exclusion, and final inclusion were not available and were not retrospectively reconstructed.

## 3. Continuous Glucose Monitoring: Principles, Performance and Clinical Utility

CGM was developed to overcome important limitations of conventional self-monitoring of blood glucose. Capillary glucose measurements provide isolated values obtained at predefined or patient-selected time points and consequently offer only a partial representation of the glycemic status. Fluctuations occurring between measurements, including asymptomatic nocturnal hypoglycemia, may therefore remain undetected. In contrast, CGM provides repeated measurements over prolonged periods, allowing glucose concentrations to be evaluated as dynamic profiles and interpreted in relation to factors such as food intake, physical activity, sleep, and pharmacological treatment.

### 3.1. Measurement Principle and Analytical Performance

Most CGM systems rely on minimally invasive enzyme-based electrochemical sensors positioned in the subcutaneous tissue, commonly at the upper arm or abdomen [35]. Unlike conventional glucometers, which measure glucose in capillary blood, these devices determine glucose concentrations in the interstitial fluid. Because glucose diffuses between the vascular and interstitial compartments, interstitial concentrations closely reflect blood glucose levels. However, the two compartments do not change simultaneously. Glucose diffusion into the interstitial space produces a physiological delay, generally within the range of 5–15 min [22,33,35,36]. This delay is not fixed and becomes particularly relevant during rapid changes in glucose concentration, such as in the postprandial period or during exercise. The observed delay reflects several components, including the physiological transport of glucose from blood to the interstitial compartment, sensor response time and signal processing, while discrepancies between blood and sensor glucose may become more pronounced when glucose concentrations are changing rapidly [37]. Modern CGM systems use signal-processing algorithms together with information on the direction and rate of glucose change to improve the interpretation of glucose trends and support predictive alerts, although these approaches do not eliminate the underlying physiological blood-to-interstitial delay [37,38].

The analytical accuracy of CGM systems is commonly assessed using the mean absolute relative difference (MARD), which expresses the relative difference between sensor-derived glucose concentrations and corresponding reference measurements [22]. Lower MARD values indicate closer agreement between sensor-derived glucose concentrations and the reference method, reflecting greater analytical accuracy. A difference of approximately 10% between CGM and reference measurements has been considered compatible with effective glucose monitoring without systematic reliance on conventional self-monitoring [16]. Due to the electrochemical principle of detection, redox-active medications such as acetaminophen, hydroxyurea or ascorbic acid can interfere with glucose detection by CGM systems. In the case of new-generation Dexcom sensors (G6 or G7), a permeselective membrane limits acetaminophen interference, allowing accurate readings at standard doses of 1 gram every 6 h, while higher supra-therapeutic doses can lead to significant bias [39,40]. Hydroxyurea, however, continues to interfere with these sensors, producing falsely elevated readings, and the manufacturer advises against using the system for treatment decisions in patients receiving hydroxyurea [41,42]. In contrast, FreeStyle Libre sensors rely on a different detection principle: glucose oxidase is “wired” to the electrode via an osmium-based redox polymer, which allows operation at a low working potential (+40 mV vs. Ag/AgCl) [43]. This design minimizes interference from acetaminophen and hydroxyurea at therapeutic concentrations, but the sensors remain susceptible to ascorbic acid (vitamin C) [41]. For earlier generations (FreeStyle Libre 2 and FreeStyle Libre 3), the manufacturer warned that ascorbic acid intake exceeding 500 mg/day could affect sensor readings. Following a modification of the sensor, the current FreeStyle Libre 2 Plus and FreeStyle Libre 3 Plus sensors are affected only by intake exceeding 1000 mg/day [44,45,46,47].

The clinical accuracy of CGM systems is usually evaluated using error grids, such as the Clarke, Parkes (Consensus) or Surveillance error grids. In this approach, CGM-derived glucose values are plotted against paired reference measurements. Each grid is divided into distinct zones, and the clinical risk associated with a certain discrepancy is inferred from the zone into which each data pair falls [48].

Calibration represents an additional determinant of sensor performance. Depending on the device, calibration may be performed during manufacturing or may require capillary glucose measurements following sensor insertion. Factory-calibrated systems reduce the procedural requirements for users, whereas systems requiring manual calibration depend on appropriate technique and adherence to the recommended calibration schedule [49].

Sensor characteristics also vary between systems. Standard subcutaneous sensors are generally placed on the upper arm or abdomen and may remain in use for approximately 10–14 days, depending on the device. In contrast, fully implanted sensors based on fluorescent sensing technology can remain subcutaneously positioned for considerably longer periods [33,50].

### 3.2. CGM Modalities and Patient Access to Glucose Information

CGM systems can be distinguished according to whether glucose information is available to the individual during sensor wear. In blinded or professional CGM, glucose measurements are recorded without being displayed to the user and are subsequently downloaded and interpreted by healthcare professionals. This approach is particularly useful for obtaining objective glucose profiles under free-living conditions because participants cannot modify their eating or physical activity behavior in response to real-time glucose information [29].

Patient-accessible monitoring includes real-time CGM (rtCGM) and intermittently scanned CGM (isCGM). rtCGM automatically transmits glucose measurements to a receiver or compatible device and may generate alerts in response to abnormal glucose concentrations or predicted hypoglycemia. isCGM, also referred to as flash glucose monitoring, requires the user to scan the sensor to retrieve stored glucose information [51].

The distinction between these modalities is clinically relevant. A 2026 meta-analysis including 30 randomized controlled trials found that patient-accessible CGM significantly reduced HbA1c overall, although the magnitude of the effect differed according to monitoring modality. rtCGM was associated with a mean HbA1c reduction of 0.65%, compared with 0.25% for isCGM. The meta-analysis also reported greater improvements in time in range (TIR), time below range (TBR), and glycemic variability with rtCGM, consistent with its capacity to provide automatically transmitted glucose information and alerts. Conversely, isCGM may offer practical advantages related to lower cost, reduced intrusiveness, and the absence of continuous alarms, thereby limiting alarm-related burden [29].

### 3.3. Clinical Utility and Glycemic Outcomes

The clinical relevance of CGM extends beyond the greater frequency of glucose measurements. Continuous acquisition of glucose data enables identification of glycemic trends, recurrent patterns, periods of hypo- and hyperglycemia, and fluctuations that may remain undetected with intermittent capillary measurements.

CGM also generates dynamic measures of glycemic control, including TIR, time in tight range (TITR), and indices of glycemic variability such as mean amplitude of glycemic excursions, standard deviation, and coefficient of variation [29].

Clinical evidence has been particularly extensive in type 1 diabetes. Studies have demonstrated improvements in glycemic control, including reductions in HbA1c and hypoglycemic events, among individuals using CGM compared with conventional self-monitoring [52,53]. In insulin-treated patients at increased risk of hypoglycemia, continuous monitoring was associated with a 72% reduction in hypoglycemic episodes [54]. Benefits extending beyond biochemical outcomes have also been reported, including reduced fear of hypoglycemia and improved quality of life [55].

Evidence also supports CGM use in insulin-treated type 2 diabetes. In a 52-week study, CGM was associated with a reduction of more than one percentage point in mean HbA1c compared with conventional glucose monitoring [56]. More broadly, systematic reviews and meta-analyses of randomized controlled trials evaluating patient-accessible CGM within lifestyle and nutritional interventions have reported reductions in HbA1c of approximately 0.28–0.48%, together with improvements in TIR, body weight, body mass index, fasting glucose, and systolic blood pressure [19,29,31].

### 3.4. From Continuous Measurement to Actionable Feedback

The effectiveness of CGM depends not only on sensor accuracy but also on how glucose information is interpreted and used. Patient interaction with CGM data can range from passive monitoring to reactive, active, proactive, and educational use. Reactive use involves responding to device-generated alerts, whereas active and proactive use requires individuals to interpret current measurements, graphical profiles, and glycemic trends and to adjust treatment or behavior accordingly [57]. Educational use further incorporates CGM data into interactions with healthcare professionals to identify appropriate therapeutic or behavioral modifications. Real-time visualization of glucose responses can also encourage changes in food selection, portion size and physical activity. Consequently, access to CGM data alone does not ensure clinical benefit; appropriate training, correct interpretation of measurements and alerts, adherence to sensor use, and continued professional support remain important components of effective implementation [57,58].

### 3.5. Technical Limitations and Barriers to Implementation

Despite its clinical utility, CGM remains subject to physiological, analytical, and practical limitations. The temporal difference between blood and interstitial glucose must be considered when glucose concentrations change rapidly [18,33]. Sensor performance may additionally be affected by enzymatic degradation, calibration drift, biochemical interference, and physical signal artifacts. Electrochemical sensors may be influenced by substances including paracetamol (acetaminophen), high-dose vitamin C and salicylic acid, potentially producing inaccurate glucose readings. Physical pressure on the sensor may also interfere with measurements. For example, sleeping directly on a sensor can reduce local microvascular perfusion and produce an apparent decrease in interstitial glucose, potentially resulting in false hypoglycemia alerts and sleep disruption [16,18,29,59].

Several technological strategies are being investigated to improve sensor stability and reduce interference, including enzyme immobilization approaches, non-enzymatic sensing materials and anti-interference oxygen-scavenging systems [16]. Beyond analytical limitations, economic and psychological factors may restrict wider CGM adoption.

Device cost and limited reimbursement remain important barriers, particularly for individuals without insulin-treated diabetes.

### 3.6. Toward Broader Applications of Continuous Glucose Sensing

The expanding use of CGM has stimulated interest in less invasive approaches to continuous glucose monitoring. Several alternative strategies have been investigated, including reverse iontophoresis, impedance spectroscopy, near-infrared and Raman spectroscopy, thermal emission spectroscopy, optical coherence tomography, sonophoresis, and polarimetry. Other proposed platforms include earlobe-based systems and glucose-sensitive biosensors incorporated into eyeglasses, contact lenses, and temporary smart tattoos [18,33].

Although these approaches differ considerably in their sensing principles and technological maturity, they illustrate ongoing efforts to extend CGM beyond conventional subcutaneous sensors. The increasing availability of continuous glucose data also creates opportunities for applications extending beyond conventional diabetes management.

By capturing postprandial glucose responses under real-world conditions, CGM enables the characterization of individual metabolic responses to food intake and lifestyle behaviors. These dynamic response patterns can contribute to individual metabolic phenotyping and provide a basis for extending continuous glucose monitoring from conventional glycemic management toward individualized nutritional assessment and precision-nutrition strategies [60].

## 4. CGM Enables Precision Nutrition

### 4.1. Foundations of Precision Nutrition and the Role of CGM

Conventional dietary assessment continues to depend largely on self-reported information obtained through instruments such as food diaries and 24-hour dietary recalls. Although widely used, these approaches impose a considerable burden on individuals and are vulnerable to poor adherence, recall errors, and systematic inaccuracies in the estimation of portion sizes, energy intake, and macronutrient consumption. Their limitations become particularly relevant in the context of the pronounced inter-individual heterogeneity observed in PPGRs. The observation that identical foods can generate markedly different glucose responses among individuals has contributed to increasing interest in individualized nutritional strategies [26,28]. However, particularly in individuals without diabetes, interindividual differences in postprandial glucose responses do not necessarily indicate pathological glucose regulation or establish that modification of these responses will provide clinical benefit.

Precision nutrition, closely related to personalized nutrition, has emerged in response to this heterogeneity. It represents a technology-supported approach in which biological, metabolic, genetic, environmental, and behavioral characteristics are integrated to develop dietary strategies tailored to the individual, with the objective of preventing or managing chronic disease, while promoting overall health [17,19]. The International Society of Nutrigenetics/Nutrigenomics (ISNN) conceptualizes precision nutrition at three progressive levels. The first comprises conventional nutritional recommendations tailored to population groups according to characteristics such as age, sex, and social determinants. The second incorporates individual phenotypic information, including anthropometric, biochemical, metabolic, and physical-activity characteristics. The third represents genotype-directed nutrition based on rare or common genetic variation [61].

The development of precision nutrition has been facilitated by advances in omics technologies, particularly genomics, epigenomics, proteomics, and metabolomics, which enable the simultaneous characterization of large numbers of biological molecules in matrices such as blood, serum, and feces. Among these approaches, metabolomics is particularly dynamic because, unlike the relatively stable genome, the metabolome reflects the combined influence of genetic characteristics and environmental exposures. Distinct metabolic profiles have been associated with obesity and type 2 diabetes, including alterations in lactate, glycolytic metabolites, branched-chain and aromatic amino acids, and long-chain fatty acids [8].

Within this broader precision-nutrition framework, CGM provides a means of capturing the individual’s glycemic response to food and lifestyle exposures in real time. Rather than characterizing foods solely according to their expected population-level effects, CGM makes it possible to observe the magnitude, duration, and temporal characteristics of glucose excursions at the individual level. Consequently, CGM-derived data can contribute to metabolic phenotyping by providing a dynamic phenotypic layer that connects dietary exposure with an individual’s metabolic response and can subsequently be combined with other biological and behavioral information.

**Figure 1:**
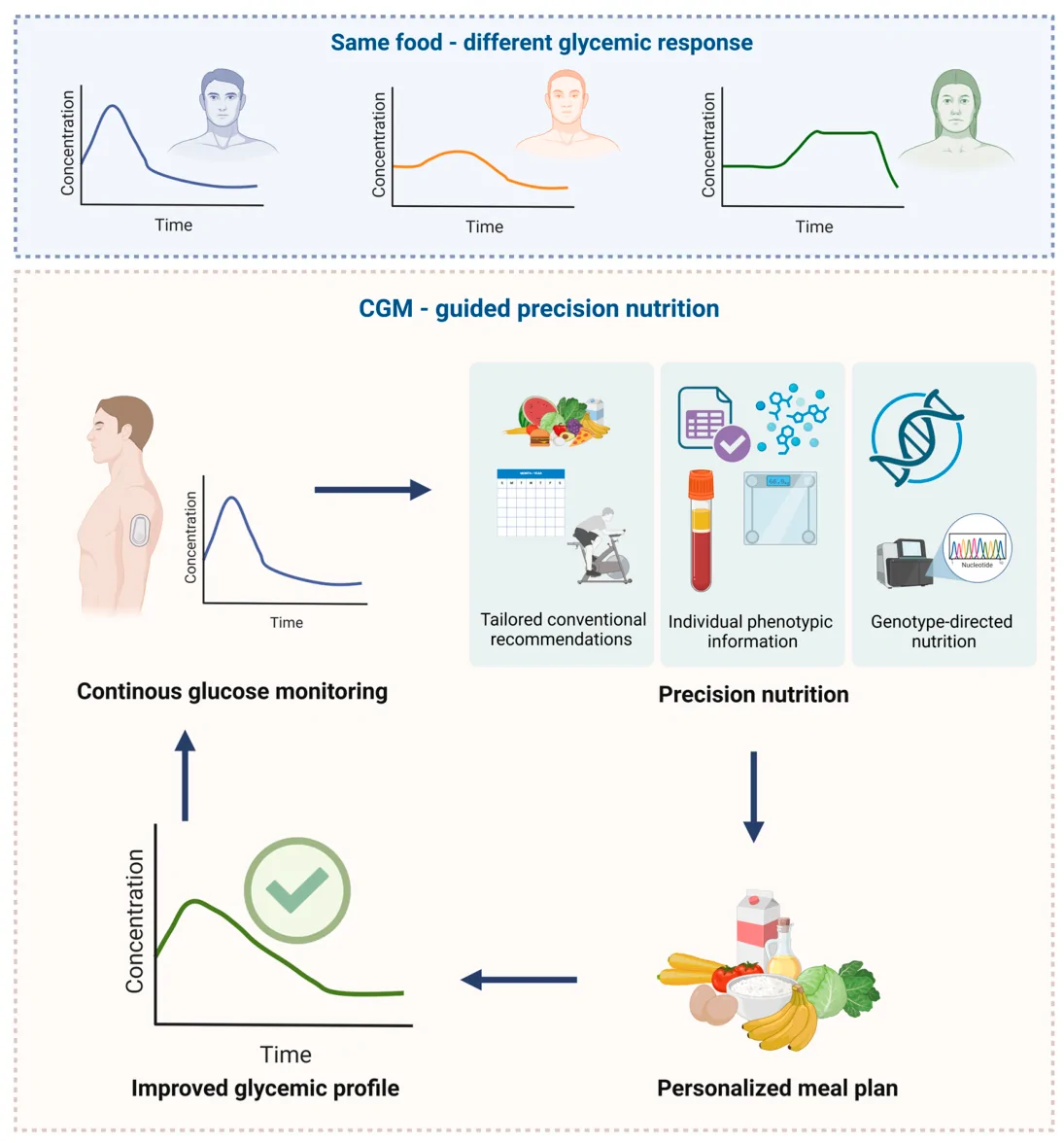
Conceptual framework of CGM-guided precision nutrition. Created with BioRender.com.

This conceptual role of CGM within precision nutrition, from the characterization of individual glycemic responses to their integration with other phenotypic and biological information for personalized dietary guidance, is illustrated in Figure 1.

### 4.2. CGM-Derived Metabolic Phenotypes

Currently, diabetes and prediabetes are diagnosed using static measures, such as fasting blood glucose or HbA1c, which may fail to capture dynamic fluctuations in glycemic profiles. CGM has been proposed as a means of characterizing these fluctuations and improving the assessment of diabetes risk in the prediabetic or healthy population, although its added value remains debated and the available evidence is conflicting. In a study of 57 healthy volunteers monitored with CGM, Hall et al. demonstrated that individuals classified as normoglycemic by standard criteria nonetheless exhibited substantial glycemic variability, with glucose concentrations falling within the prediabetic and diabetic ranges 15% and 2% of the time, respectively [32]. Using spectral clustering, the authors developed an algorithm that stratified participants into three “glucotypes” characterized by low, moderate, and severe glycemic variability. However, when Hulman et al. applied this algorithm to an independent cohort to assess its generalizability and interpretability, they found that the glucotype of 107 of 116 individuals could be correctly predicted from mean CGM glucose alone, suggesting that glucotype classification provides limited additional information or that the data obtained by Hall et al. cannot be transferred to other datasets due to overfitting [62].

The reproducibility of metabolic phenotypes is likewise a matter of debate. Hall et al. reported good intra-individual reproducibility of PPGRs when participants consumed the same meal on two occasions [32]. In contrast, an analysis of published data from two clinical research protocols found that the reliability of postprandial CGM responses to duplicate multicomponent meals was poor, with within-subject variability in responses to identical meals approximately as large as the variability observed across different meals (intraclass correlation coefficients (ICCs) of 0.28 and 0.17 for the Abbott and Dexcom devices, respectively) [63].

Howard et al. examined whether two CGM devices worn simultaneously would yield concordant data. Collecting 27,489 paired measurements from Dexcom G4 Platinum and Abbott FreeStyle Libre Pro sensors in 16 adults without diabetes, they observed discordance between the two devices in within-subject meal rankings. Notably, the devices differ in sampling frequency (5-min versus 15-min intervals), which may influence the identification of glycemic peaks and the calculation of the iAUC [64].

In summary, the evidence in this area remains inconsistent. Although consensus clinical targets for CGM have been established for the management of diabetes, no comparable consensus exists for its application in precision nutrition, which therefore warrants further investigation [65].

### 4.3. Determinants of Postprandial Glycemic Variability

The postprandial glucose curve is not determined exclusively by carbohydrate intake. Its amplitude, duration, and overall configuration reflect the interaction of dietary composition, food structure and processing, behavioral factors, and meal timing [1].

Although carbohydrates represent the principal dietary determinant of the immediate increase in postprandial glucose, their glycemic effect can be substantially modified when they are consumed as part of mixed meals. The addition of dietary fiber, protein, and healthy fats can modify the magnitude and duration of PPGRs, with mixed meals generally producing lower and more prolonged glucose excursions than carbohydrate-only meals [1,16].

The physical characteristics of food and the way it is processed or prepared provide an additional source of variability. Processes that increase digestibility, such as gelatinization and milling of whole wheat, can increase postprandial glucose responses. In contrast, cooking followed by cooling, as described for foods including potatoes and chickpea pasta, promotes the formation of resistant starch and can consequently attenuate the postprandial glycemic response [16,66,67].

Meal sequence also represents a modifiable determinant of glycemic exposure. Consuming fiber- and protein-containing foods before carbohydrate-rich foods can reduce post-meal glucose excursions, providing a relatively simple behavioral strategy for modifying the shape of the postprandial curve [18].

Beyond individual meals, glycemic variability is also associated with broader lifestyle and circadian behaviors. Sedentary behavior and shorter sleep duration have been linked to greater glycemic variability, whereas earlier evening meals and shorter daily eating windows have been associated with improved glycemic control in free-living settings. Taken together, these observations emphasize that CGM data should be interpreted within the wider context of meal composition, food preparation, eating sequence, physical activity, sleep, and meal timing, rather than as an isolated response to carbohydrate ingestion [59].

### 4.4. Machine Learning and Individualized Prediction of Postprandial Response

The integration of CGM data with computational modeling has expanded the role of CGM beyond the retrospective characterization of glucose excursions toward the prediction of individualized metabolic responses.

In this framework, CGM-derived glycemic profiles can be analyzed together with complementary biological, clinical, dietary, and behavioral variables, including anthropometric characteristics, biochemical measurements, habitual dietary intake, physical activity, and gut microbiome features. Machine-learning and statistical modeling approaches can subsequently identify complex relationships among these variables and estimate an individual’s PPGR to specific dietary exposures. A demonstration of this concept was provided by Zeevi et al. in 2015 [12]. The investigators developed a stochastic gradient boosting regression model based on sequentially trained decision trees that incorporated blood parameters, anthropometric characteristics, dietary habits, physical activity, and gut microbiome composition. Rather than assuming a uniform glycemic effect of a given food across individuals, the model was designed to predict individualized two-hour PPGRs, quantified using the incremental area under the glucose curve (iAUC). Subsequent investigation by Mendes-Soares et al. demonstrated that the predictive model developed in the Israeli cohort could predict postprandial glycemic responses in an independent Midwestern U.S. cohort, supporting the generalizability of the predictive framework across populations [68].

Another successful validation example comes from the study of Berry et al., who recruited a cohort of 1002 UK volunteers and validated their findings in an independent US cohort of 100 people, with a model predicting glycemic responses at r = 0.77 [14]. According to Popova et al., this level of predictive accuracy may approach the upper limit imposed by the inherent variability of PPGRs of 0.7–0.77 reported by Zeevi et al. [12,69].

However, the importance of microbiome-derived variables in PPGR prediction remains uncertain. Methodological concerns have been raised regarding early personalized prediction studies, including the interpretation of within- versus between-person variability and whether complex individualized algorithms offer meaningful advantages over simpler approaches to dietary assessment [70]. PPGRs are influenced by multiple characteristics of the individual and the consumed meal, and more recent evidence indicates that microbiome information may add predictive value but does not necessarily account for a large proportion of the overall predictive performance [69]. Thus, microbiome-derived features are better viewed as one component of a multifactorial predictive framework rather than as a primary determinant of individualized glycemic responses.

Within this evolving field, particular attention has been directed toward determining whether microbiome-derived information improves the prediction of PPGR beyond conventional host and dietary characteristics. This question was further examined in a two-week cohort study involving 550 adults, in which participants consumed standardized meals with varying combinations of macronutrients and dietary fiber under free-living conditions. The resulting glycemic data demonstrated substantial interindividual heterogeneity and were incorporated into a multilevel mixed-effects regression model together with biological, nutritional, and behavioral variables. Importantly, functional characteristics of the gut microbiome contributed significantly to the observed variation in PPGRs beyond information provided by conventional clinical, anthropometric, and dietary factors. These findings further support the concept that the gut microbiome represents an additional source of information for individualized prediction rather than merely an accompanying biological measurement [71].

Gut microbiota may contribute to interindividual differences in glucose metabolism through microbial metabolites and their interactions with host metabolic pathways. *Akkermansia muciniphila* and *Faecalibacterium prausnitzii* are among the bacterial species that have been associated with glucose-related outcomes, although evidence that short-chain fatty acids (SCFAs) mediate these associations remains limited [72]. In a randomized proof-of-concept study in overweight or obese insulin-resistant adults, supplementation with *A. muciniphila* provided preliminary evidence of improved insulin sensitivity [73]. More broadly, microbiota-derived SCFAs have been implicated in pathways relevant to glucose homeostasis and insulin resistance, including intestinal barrier function, insulin and gut-hormone secretion, and immune-inflammatory regulation [74]. These observations provide biological plausibility for incorporating microbiome-derived features into predictive models, while not establishing the microbiome as a causal determinant of individual postprandial glycemic responses.

From a precision-nutrition perspective, such predictive models provide a potential framework for translating baseline microbiome and host characteristics into food-specific recommendations. Instead of requiring continuous observation of the glycemic response to every dietary exposure, an established model could be used to estimate the likely metabolic effect of individual foods and classify them according to predicted positive, neutral, or negative effects. Accordingly, sufficiently validated predictive models could potentially reduce dependence on prolonged or repeated CGM use while supporting individualized dietary guidance [29].

The incorporation of microbiome information into computational nutrition is not limited to the prediction of PPGR. A complementary approach has been implemented within the European *Stance4Health* project through AGREDA, an extended reconstruction of dietary metabolism in the human gut microbiota algorithm. Here, genome-scale metabolic models of the gut microbiota are used to estimate how specific dietary choices may influence microbial metabolic activity and the production of potentially beneficial postbiotic metabolites. Whereas PPGR models use microbiome and host characteristics to improve predictions of individual glycemic responses, this approach focuses more directly on modeling the metabolic consequences of diet–microbiome interactions. Together, these strategies illustrate how computational precision nutrition can integrate both host metabolic responses and microbial functionality when evaluating the potential effects of dietary exposures [75,76].

Alongside the development of increasingly complex predictive models, the availability of standardized multimodal datasets has become important for their evaluation and reproducibility. The open-access CGMacros dataset was developed for this purpose by combining CGM measurements obtained with Abbott FreeStyle Libre Pro and Dexcom G6 Pro devices with meal macronutrient information, smartwatch-derived physical activity data, food photographs, and microbiome profiles. Because the CGM devices operated at different sampling frequencies, linear interpolation was used to harmonize sensor outputs into standardized minute-by-minute glucose data. The predictive utility of the dataset was subsequently evaluated using an Extreme Gradient Boosting (XGBoost) model to estimate two-hour postprandial responses. The model achieved Pearson correlation coefficients of r = 0.89 for absolute area under the curve (AUC) and r = 0.64 for iAUC, indicating predictive performance comparable with that obtained in substantially larger previous cohorts. Thus, CGMacros provides an example of how heterogeneous CGM, dietary, wearable, and microbiome data can be integrated within a common computational framework for individualized PPGR prediction [2]. It should be noted, however, that the CGMacros dataset comprises data from only 45 participants, which increases the risk of overfitting in models trained on these data and underscores the need for larger datasets to further validate these findings.

Most computational approaches described above follow a forward prediction paradigm: information about the individual and the consumed meal is used to predict the subsequent glycemic response. A conceptually distinct application reverses this relationship. This so-called inverse metabolic problem seeks to infer characteristics of a consumed meal from the glucose response that follows it. In other words, rather than asking how a known meal is expected to affect glucose, the model attempts to reconstruct dietary intake from the configuration of the postprandial glycemic curve [2]. This inverse modeling strategy introduces a different potential role for CGM within precision nutrition: CGM data may not only be used to predict metabolic responses to recorded dietary intake but may also contribute to reconstructing aspects of dietary intake itself. Such an approach could ultimately reduce reliance on burdensome manual dietary reporting and facilitate more automated nutritional assessment.

### 4.5. Clinical Outcomes of CGM-Supported Nutritional and Behavioral Interventions

Beyond its analytical and predictive functions, CGM has been evaluated as a practical component of nutritional and lifestyle interventions. The continuous visualization of glucose responses provides individuals with immediate information regarding the metabolic consequences of dietary choices and behaviors, potentially transforming glucose measurements into actionable feedback. Evidence from randomized trials and meta-analyses indicates that CGM-supported interventions can influence glycemic outcomes as well as selected cardiometabolic parameters.

The clinical evidence summarized here derives from heterogeneous study designs and should therefore not be interpreted as representing equivalent levels of evidence. It includes systematic reviews and meta-analyses of randomized controlled trials (RCTs), individual randomized studies, and an uncontrolled pilot feasibility study [24,29,30,31,77,78]. The studies also differ in sample size, follow-up duration, comparator conditions, and intervention composition. Importantly, in several studies CGM was incorporated into multicomponent nutritional or behavioral interventions, whereas in others it was used primarily to quantify glycemic outcomes. The intervention evidence summarized in Table 1 is predominantly derived from individuals with type 2 diabetes, reflecting the greater maturity of the clinical evidence in this population. Evidence supporting CGM-guided nutritional interventions in individuals without diabetes remains substantially more limited and is discussed separately below. These methodological characteristics, together with relevant funding and conflicts of interest, are summarized in Table 1 to facilitate critical interpretation of the available evidence (Table 1).

**Table 1:** Selected clinical evidence on CGM-supported nutritional and behavioral interventions: study characteristics, outcomes, methodological considerations and funding/COI.

Study	Population/*N*	Design/Follow-Up	Intervention/Comparator	Main Findings	Key Limitations	Funding/COI
Bannuru et al. [31]	21 RCTs; 2734 adults with T2D; 20 RCTs meta-analyzed.	Systematic review/meta-analysis of RCTs; 2–12 months; RoB 2 + GRADE.	CGM + nutrition/lifestyle education or feedback vs. standard care/education.	HbA1c −0.46%; TIR +7.18%; TAR −7.32%; weight −2.06 kg.	Moderate certainty for major outcomes; substantial heterogeneity (HbA1c I^2^ = 88%; weight I^2^ = 90%). Multicomponent interventions limit attribution to CGM alone.	Partly supported by an unrestricted Abbott Diabetes Care grant to ADA; several authors reported industry relationships.
Sebastian et al. [29]	30 RCTs; 3133 adults with T2D; 19 rtCGM, 11 isCGM; 17 included insulin-treated participants.	Systematic review/meta-analysis of RCTs; follow-up ≤ 14 months.	Patient-accessible rtCGM or isCGM vs. SMBG/usual care.	HbA1c −0.48% (rtCGM −0.65%; isCGM −0.25%); TIR +10.76%; weight −3.26 kg; SBP −3.47 mmHg; non-HDL −9.31 mg/dL.	Heterogeneous CGM modalities/treatment populations; five trials used older-generation rtCGM. Longer cardiometabolic-outcome RCTs needed.	No funding or competing interests reported.
Richardson et al. [24]	25 RCTs; 2996 participants; 17/25 (68%) T2D; other populations included T1D, gestational diabetes and obesity.	Systematic review/meta-analysis of RCTs; 2–52 weeks (median 15); RoB 2.	CGM-based feedback for behavior changes vs. comparator without CGM feedback; all interventions multicomponent.	HbA1c −0.28% (95% CI −0.42 to −0.15); TIR +7.4% (2.0 to 12.8); no significant weight/BMI effect.	High heterogeneity (HbA1c I^2^ = 88%; TIR I^2^ = 80.5%); predominantly T2D. Multicomponent designs prevent isolation of CGM effect.	No review funding; 44% of included RCTs reported CGM-industry COIs; review authors reported relevant consulting relationships.
Oliveira et al. [30]	*N* = 121 adults with T2D; 53% women; mean age 64 years.	Remote parallel-group RCT; 12 weeks; intention-to-treat.	Low-carbohydrate vs guideline-based low-fat breakfast; blinded FreeStyle Libre Pro used during first/last 14 days.	Within low-carb group HbA1c −0.3%; between-group difference −0.2% (95% CI −0.4 to 0.0; *p* = 0.06). Several CGM metrics favored low carb.	Tests breakfast composition, not CGM efficacy; CGM was primarily an outcome measure. Primary between-group HbA1c difference was not significant.	Funded by Egg Nutrition Center and Egg Farmers of Canada; no CGM-manufacturer funding reported.
Martens et al. [77]	*N* = 72 adults with T2D; CGM alone *n* =31; CGM + food logging *n* = 41.	Randomized prospective four-center study; 6 months.	CGM alone vs CGM + food-logging app; both groups received guided CGM-based education.	TIR 46% → 71% at 3 months → 72% at 6 months; HbA1c −1.3%; weight −7 lb. Between-group TIR change: *p* = 0.36 (3 mo), *p* = 0.06 (6 mo).	No non-CGM control; both groups received education. Six-month duration and unmeasured self-care behaviors limit interpretation.	Sponsored by Abbott Diabetes Care; several authors reported industry relationships.
Oser et al. [78]	*N* = 17 adults with recently diagnosed T2D; 2 dropouts.	Uncontrolled pilot feasibility study; 6-week intervention; 3-month follow-up.	GEM: CGM + dietary/physical-activity guidance, activity monitor and motivational texts; no control group.	HbA1c 8.0% → 6.2% (−1.8%; *p* < 0.001); 66.7% met study remission criterion (HbA1c < 6.5%).	Very small uncontrolled sample; short follow-up; multicomponent intervention prevents attribution to CGM alone and limits generalizability.	Abbott supplied CGM devices; one author reported advisory roles with Dexcom and Cecilia Health.

Abbreviations: ADA, American Diabetes Association; BMI, body mass index; CI, confidence interval; CGM, continuous glucose monitoring; COI, conflict of interest; GEM, glycemic excursion minimization; GRADE, Grading of Recommendations Assessment, Development and Evaluation; HbA1c, glycated hemoglobin; isCGM, intermittently scanned continuous glucose monitoring; RCT, randomized controlled trial; RoB 2, revised Cochrane Risk of Bias tool; rtCGM, real-time continuous glucose monitoring; SBP, systolic blood pressure; SMBG, self-monitoring of blood glucose; T1D, type 1 diabetes; T2D, type 2 diabetes; TAR, time above range; TIR, time in range.

Three systematic reviews and meta-analyses of RCTs reported improvements in selected glycemic outcomes associated with CGM-supported interventions [24,29,31]. Bannuru et al. reported reductions in HbA1c and body weight and improvements in TIR and TAR, with moderate certainty of evidence for these outcomes. However, substantial heterogeneity was observed for several outcomes, and many of the included studies incorporated CGM within nutritional education or broader lifestyle interventions, limiting attribution of the observed effects to CGM alone [31]. Sebastian et al. similarly reported improvements in HbA1c, TIR, body weight, systolic blood pressure, and non-HDL cholesterol, although the included trials differed in CGM modality, treatment population, and device generation [29]. Richardson et al. reported modest improvements in HbA1c and TIR, but not in body weight or BMI. This review also showed substantial heterogeneity, and all included interventions were multicomponent, further limiting conclusions regarding the independent contribution of CGM [24].

The individual randomized studies provide more context-specific evidence. Oliveira et al. compared a low-carbohydrate with a guideline-based low-fat breakfast in 121 adults with type 2 diabetes over 12 weeks. Although several CGM-derived glycemic measures favored the low-carbohydrate intervention, the prespecified between-group difference in HbA1c did not reach conventional statistical significance (*p* = 0.06). Moreover, CGM was used primarily to quantify glycemic outcomes rather than constituting the intervention itself; therefore, this study primarily provides evidence regarding dietary composition rather than the independent effectiveness of CGM [30]. In the 6-month randomized study by Martens et al., TIR, HbA1c, and body weight improved among participants using CGM with guided education, with or without an additional food-logging application. However, both randomized groups received CGM and guided education, and there was no non-CGM control group. Consequently, the observed improvements cannot be attributed specifically to CGM compared with usual care, and between-group differences in TIR change were not statistically significant [77].

The remission findings reported by Oser et al. warrant particularly cautious interpretation. In this uncontrolled pilot feasibility study, 17 adults with recently diagnosed type 2 diabetes participated in a multicomponent intervention incorporating CGM feedback, dietary carbohydrate reduction, increased physical activity, activity monitoring, and motivational support. HbA1c decreased substantially, and 66.7% of participants met the study-defined remission criterion at follow-up. However, the very small sample, absence of a control group, short follow-up, and multicomponent nature of the intervention preclude attribution of the observed effects specifically to CGM and substantially limit generalizability. These preliminary findings require confirmation in larger controlled studies [78].

It should be emphasized that much of the clinical intervention evidence summarized above derives from individuals with type 2 diabetes, whereas evidence supporting CGM-guided nutritional and lifestyle interventions in individuals without diabetes remains considerably more limited. Interpretation of CGM-derived metrics in this population also requires caution. Preliminary reference ranges for glycemic variability have been proposed in healthy populations, including for metrics such as mean amplitude of glycemic excursions (MAGE), but clinically validated benchmarks and scoring procedures for CGM measures in people without diabetes remain insufficiently established. Consequently, postprandial glucose excursions observed in individuals without diabetes should not automatically be interpreted as clinically relevant abnormalities or as targets requiring dietary intervention. Evidence regarding long-term clinical benefit is also limited. Systematic reviews examining CGM in non-diabetic populations have highlighted the scarcity of available intervention studies and the uncertainty regarding effects on cardiovascular outcomes. Therefore, although CGM can characterize individual glucose responses and may support lifestyle feedback, further prospective evidence is required to establish whether targeting these responses improves long-term clinical outcomes in individuals without diabetes [28,34,79].

### 4.6. From Glucose Data to Personalized Dietary Feedback

For precision nutrition to operate outside controlled research environments, complex CGM signals must be converted into information that users can readily interpret. Commercial digital-health platforms have begun to integrate glucose measurements with dietary records to generate individualized scores or recommendations. Vively represents one example of this approach.

The Vively platform, for example, translates meal and CGM information into two distinct scores. Its Diet Quality Score, expressed on a 0–10 scale, evaluates logged meals according to factors including macronutrient distribution, degree of food processing, and alcohol consumption. A separate Diet Glucose Score, also ranging from 0 to 10, is calculated from the two-hour postprandial glucose profile. This score incorporates the area under the glucose curve, time within the target range, and peak glucose concentration to quantify the compatibility of a meal with the individual’s observed metabolic response [19]. These findings should nevertheless be interpreted as platform-specific observational evidence rather than as evidence of effectiveness across digital precision-nutrition systems. The scoring algorithms and user-engagement features are specific to the Vively platform, and the observational design does not establish that the reported engagement patterns or behavioral responses would generalize to other CGM-based digital interventions. The study used deidentified data provided by Vively under a data-sharing agreement; according to the authors, Vively had no role in the study design, data analysis or interpretation, or the decision to submit the manuscript.

However, the effectiveness of digital precision-nutrition systems depends heavily on sustained user engagement. Real-world observational data from 7647 users of the Vively application showed that 91.7% engaged in food logging. Logging behavior was nevertheless strongly dependent on active CGM use. During periods of sensor wear, food was logged on an average of 63.2% of days, whereas logging fell to only 2.4% of days during breaks without an active sensor. This marked decline illustrates a passive tracking paradox, in which engagement with dietary self-monitoring appears to be reinforced by the availability of immediate physiological feedback and substantially diminishes when that feedback is removed. Integration with other wearable technologies may partially strengthen engagement. Users who synchronized third-party wearable devices, such as smartwatches, recorded food on 45% more days and maintained CGM use for 32% longer periods. In addition, each 1 mmol/L increase in mean baseline glucose was associated with 4% fewer food-logging days (incidence rate ratio (IRR) 0.96, 95% confidence interval (CI) 0.94–0.98), while higher baseline glucose was simultaneously associated with longer CGM wear (IRR 1.15, 95% CI 1.13–1.17) [19].

### 4.7. Psychological and Behavioral Limitations of Continuous Metabolic Feedback

Despite its capacity to support dietary personalization, continuous access to glucose information may also generate unintended psychological consequences. Continuous metabolic feedback has therefore been described as a potential double-edged sword. Qualitative evidence indicates that persistent attention to glucose values may alter the user’s relationship with food, generating information overload and frustration while reducing reliance on intuitive or mindful eating behaviors as individuals attempt to maintain ideal glucose profiles.

These concerns are supported by mixed-methods evidence. In a study of 56 adults with and without diabetes who were not using insulin, 68% reported fear of type 2 diabetes when observing elevated glucose values. CGM-related distress was also associated with individual characteristics: younger age and obesity were associated with greater distress, while agreeableness was associated with greater fear and unhappiness in response to elevated glucose levels. Higher eating-disorder symptoms, assessed using the Eating Disorder Examination Questionnaire-Short (EDE-QS), were also associated with distress related to the appearance of the CGM sensor. Notably, despite these psychological burdens, 89% of participants reported positive changes in dietary intake and/or physical activity, indicating that perceived behavioral benefits and psychological distress may coexist [80].

Individual psychological characteristics may further influence vulnerability to CGM-related distress. Agreeableness was positively correlated with fear (r = 0.42) and unhappiness (r = 0.36) in response to elevated glucose readings. In addition, symptoms of eating disorders assessed using the Eating Disorder Examination Questionnaire-Short (EDE-QS) were associated with distress concerning the physical appearance of the sensor (r = 0.40).

These findings highlight the importance of considering psychological responses alongside metabolic outcomes when CGM is incorporated into personalized nutrition strategies [19]. It is essential to consider and implement, where possible, strategies that could potentially reduce the psychological burden of CGM. For example, a nurse-led intervention demonstrated that five daily 90 min sessions integrating education, skill training, self-management and coping strategies in addition to CGM led to a decrease in anxiety and depression scores reported in adults with type 1 diabetes. Further strategies may include personalizing alarm thresholds to mitigate alarm fatigue, and offering psychological support, particularly during the initial period of sensor use, when the volume of glucose data may feel most overwhelming. Nutritional support from a dietitian or nutritionist may also be beneficial. Delivering these interventions both in person and remotely could improve accessibility for a wider population [81].

### 4.8. Automation of Dietary Assessment and Emerging Wearable Technologies

A persistent limitation of nutritional monitoring is the requirement for individuals to manually document food consumption. Automated detection of ingestive activity therefore represents an important technological objective, particularly if precision-nutrition systems are to operate with minimal user burden. A scoping review encompassing 19 publications representing 17 studies found that CGM-based algorithms can identify eating events with sensitivities exceeding 90%. Nevertheless, the detection of food intake generally occurs with a delay of approximately 9–45 min. This temporal delay limits the suitability of CGM-based meal detection for interventions intended to modify behavior while a meal is being consumed. In particular, it complicates the implementation of Just-In-Time Adaptive Interventions (JITAIs) during the eating episode itself. However, the same technology remains useful for retrospective identification of meals, automated dietary logging, and delivery of postprandial prompts [82].

Beyond automated dietary assessment, the increasing volume of longitudinal CGM data creates additional challenges for clinical implementation. Such datasets may be difficult to incorporate efficiently into routine clinical decision-making. Consequently, artificial intelligence (AI)-enabled clinical decision support systems (AI-CDSS) integrated into electronic health records have been identified as an important future requirement for primary care. These systems could support the interpretation of extensive longitudinal CGM datasets while optimizing clinical workflow and limiting the additional burden imposed on healthcare professionals [83].

Overall, the integration of CGM into precision nutrition extends beyond continuous measurement of interstitial glucose. CGM-derived data can contribute to individual metabolic phenotyping, support the assessment and prediction of postprandial responses, facilitate automated identification of dietary events, and be integrated with other dietary, behavioral, and biological information. However, the increasing complexity and volume of these data also highlight the need for approaches that reduce user burden and facilitate their interpretation. The development of automated dietary assessment and AI-supported data interpretation therefore represents an important direction for CGM-based precision nutrition, alongside emerging approaches to continuous metabolic monitoring [1,2].

## 5. Future Perspectives

While the technical, psychological, and accessibility-related limitations of CGM remain important considerations for its broader implementation, future developments in precision nutrition are increasingly directed toward expanding the range of measurable biomarkers and integrating multiple sources of metabolic and behavioral information.

One emerging direction involves extending wearable sensing beyond glucose as a single metabolic biomarker. Continuous ketone monitoring (CKM) has demonstrated the feasibility of continuously assessing metabolic ketosis in subcutaneous tissue using enzymatic sensing technologies [84]. Other emerging platforms target biomarkers obtainable from sweat or saliva: epidermal sensors have been investigated for monitoring vitamin C, uric acid, and tyrosine, while non-invasive mouthguard systems have been developed for continuous measurement of salivary lactate. Tooth-mounted radiofrequency tri-layer sensors have also been developed to monitor oral dietary consumption. Collectively, these technologies may broaden continuous metabolic monitoring from a glucose-focused approach toward a more multidimensional characterization of individual metabolic status [85,86,87,88].

Further progress is also expected in the sensing mechanisms underlying continuous glucose monitoring. Third-generation biosensors based on direct electron transfer (DET) are being investigated as alternatives to conventional electrochemical approaches. Enzymes such as bacterial flavin adenine dinucleotide (FAD)-dependent glucose dehydrogenase (bFADGDH) and cellobiose dehydrogenase (CDH) possess intrinsic electron-transfer components that facilitate communication between the catalytic site and the electrode without requiring externally added mediators. Such approaches may also reduce susceptibility to electrochemically active interfering compounds, including acetaminophen and ascorbic acid. In parallel, abiotic and non-enzymatic sensing strategies offer potential solutions to limitations related to enzyme stability during prolonged monitoring. These include fluorescence-based systems employing synthetic diboronic acid receptors and electrochemical sensors incorporating nanostructured materials such as MXenes and platinum- or copper-based nanoparticles [89,90,91].

Another area of development is the use of alternative biofluids for less invasive glucose monitoring. Sweat, tears, and saliva provide accessible alternatives to blood or interstitial fluid, but their substantially lower glucose concentrations and the physiological delay between glucose changes in these compartments and in blood complicate accurate interpretation. In sweat-based systems, mathematical approaches that account dynamically for physiological lag and variations in sweat rate may improve the relationship between sensor measurements and blood glucose concentrations [91,92].

Future development must also consider whether continuous sensing technologies can be implemented at greater scale. Simpler manufacturing approaches, including screen-printed and paper-based non-enzymatic sensors, have shown competitive analytical performance while offering potential advantages for lower-cost production and use in resource-limited settings [91].

Sustainability represents an additional consideration because disposable sensors and their associated components contribute to healthcare-related waste. The Green Diabetes Initiative addresses this issue through five strategies—Reduce, Reuse, Recycle, Redesign, and Re-educate—while future device development may also involve smaller devices, modified materials and designs, and greater potential for reuse or recycling of sensing components [92].

The broader translation of CGM-based precision nutrition and emerging multi-analyte sensing platforms also requires consideration of the regulatory status of the device, the associated software, and the data generated by these systems. Sensors intended for medical use are subject to applicable medical-device requirements, including U.S. Food and Drug Administration (FDA) oversight in the United States and conformity assessment under the European Union (EU) Medical Device Regulation [93,94]. Software and predictive algorithms require separate consideration, as their regulatory status depends on their intended purpose and the type of information or recommendations they provide [93]. The integration of continuous glucose data with genetic, microbiome, and other health-related information also raises data-protection requirements under the General Data Protection Regulation (GDPR) in the European Union and, where applicable, the Health Insurance Portability and Accountability Act (HIPAA) in the United States [95]. Finally, differences in performance evaluation, calibration methods, and algorithms across CGM systems may limit comparability of device readings and derived metrics, highlighting the need for greater standardization and transparent validation as these technologies are incorporated into clinical and precision-nutrition applications [95].

The future contribution of continuous metabolic monitoring to precision nutrition will therefore depend on the development of more robust, less invasive, and increasingly multidimensional sensing approaches, together with the effective integration and interpretation of the resulting data to support individualized guidance. Their broader implementation will also require addressing psychological, technical, socioeconomic, and sustainability-related challenges. Ultimately, the transition from glucose-centered monitoring toward integrated metabolic assessment may enable a more comprehensive characterization of individual metabolic responses to dietary and lifestyle exposures.

## 6. Conclusions

Continuous glucose monitoring has evolved beyond its established role in diabetes management to become an important enabling technology for metabolic phenotyping and precision nutrition. By continuously capturing glucose dynamics under free-living conditions, CGM enables the characterization of substantial interindividual variability in postprandial responses that may not be captured by conventional dietary assessment or population-level nutritional approaches alone. CGM-derived glucose patterns can provide a dynamic phenotypic layer that, when integrated with dietary, anthropometric, clinical, behavioral, and microbiome-derived information, supports the prediction of individualized glycemic responses and the translation of metabolic data into personalized dietary guidance. Computational and machine-learning approaches further expand the potential of these integrated data for individualized prediction and nutritional assessment.

Clinical studies indicate that CGM-supported nutritional and behavioral interventions can improve glycemic outcomes, although the current intervention evidence is predominantly derived from individuals with type 2 diabetes. Evidence supporting comparable clinical benefits in individuals without diabetes remains substantially more limited, and the clinical significance of targeting CGM-derived glucose fluctuations in these populations has not yet been established. Accordingly, further prospective studies are required to determine whether CGM-guided nutritional interventions in individuals without diabetes translate into sustained improvements in clinically meaningful outcomes.

Emerging sensing technologies may further extend this framework beyond glucose toward a broader characterization of individual metabolic responses. Overall, CGM represents a valuable interface between continuous physiological monitoring and individualized nutrition, with its greatest potential arising from integration within comprehensive, data-driven precision-nutrition strategies.

## Data Availability

No new data were created or analyzed in this study. Data sharing is not applicable to this article.

## Abbreviations

The following abbreviations are used in this manuscript:

AI	Artificial intelligence
AI-CDSS	Artificial intelligence-enabled clinical decision support systems
AUC	Area under the curve
bFADGDH	FAD-dependent glucose dehydrogenase
BMI	Body mass index
CDH	Cellobiose dehydrogenase
CI	Confidence interval
CGM	Continuous glucose monitoring
CKM	Continuous ketone monitoring
COI	Conflict of interest
DET	Direct electron transfer
EDE-QS	Eating Disorder Examination Questionnaire-Short
FAD	Flavin adenine dinucleotide
FDA	U.S. Food and Drug Administration
GDPR	General Data Protection Regulation
HbA1c	Glycated hemoglobin
HIPAA	Health Insurance Portability and Accountability Act
iAUC	Incremental area under the curve
ICC	Intraclass correlation coefficient
IRR	Incidence rate ratio
isCGM	Intermittently scanned continuous glucose monitoring
ISNN	International Society of Nutrigenetics/Nutrigenomics
JITAI	Just-In-Time Adaptive Intervention
MAGE	Mean amplitude of glycemic excursions
MARD	Mean absolute relative difference
PPGR	Postprandial glycemic response
RCT	Randomized controlled trial
rtCGM	Real-time continuous glucose monitoring
SCFA	Short-chain fatty acid
SBP	Systolic blood pressure
SMBG	Self-monitoring of blood glucose
T1D	Type 1 diabetes
T2D	Type 2 diabetes
TAR	Time above range
TBR	Time below range
TIR	Time in range
TITR	Time in tight range
XGBoost	Extreme Gradient Boosting
